# Supplementary material for: Effectiveness of Strategies for Nutritional Therapy for Patients with Type 2 Diabetes and/or Hypertension in Primary Care: A Systematic Review and Meta-Analysis
Source: Int J Environ Res Public Health. 2022 Apr 2;19(7):4243. doi: 10.3390/ijerph19074243 (PMC8998242; doi:10.3390/ijerph19074243)
Supplement: Supplementary file 1 [file ijerph-19-04243-s001.zip › ijerph-1581998-supplementary.pdf]

Supplementary Material

Effectiveness of strategies for nutritional therapy for patients with type 2 diabetes and/or hypertension in primary care: systematic review of randomized controlled trials

Contents:

Table S1 Search Strategy PubMed

Table S2 Excluded studies and main reason for exclusion

Figure S1 Summary of risk of bias

Figure S2 Meta-analysis of nutritional therapy for hypertension**,** sensitivity analysis without Hacihasanoğlu. **A**. effect on systolic blood pression, **B**. effect on diastolic blood pressure.

Figure S3 Meta-analysis of nutritional therapy for hypertension for primary outcome (systolic blood pressure), subgroup analysis according to length of follow-up.

Figure S4 Meta-analysis of nutritional therapy for hypertension for primary outcome (systolic blood pressure), subgroup analysis according to risk of bias. Abbreviations: SC: some concerns.

Figure S5 Meta-analysis of nutritional therapy for hypertension for primary outcome (systolic blood pressure) separating studies that presented the results as mean adjusted difference (i.e. change score) or post-intervention values

Figure S6 Meta-regression assessing follow up as modifier of the effect of nutritional therapy on HbA1c

Figure S7 Meta-regression assessing mean age and mean HbA1cs as modifiers of the effect of nutritional therapy on HbA1c

Figure S8 Meta-analysis of nutritional therapy for diabetes mellitus for primary outcome (HbA1c) without Javaid et al and Hörnstein et al.

Figure S9 Meta-analysis of nutritional therapy for diabetes mellitus for primary outcome (HbA1c), subgroup analysis according to length of follow up.

Figure S10 Meta-analysis of nutritional therapy for diabetes mellitus for primary outcome (HbA1c), subgroup analysis separating studies that presented the results as mean adjusted difference (i.e. change score) or post-intervention values.

Figure S11 Meta-analysis of nutritional therapy for diabetes mellitus for primary outcome (HbA1c), subgroup analysis according to risk of bias.

Figure S12 Meta-analysis of nutritional therapy for diabetes mellitus. Secondary outcome: systolic blood pressure.

Figure S13 Meta-analysis of nutritional therapy for diabetes mellitus without Javaid et al. Secondary outcome: systolic blood pressure.

Figure S14 Meta-analysis of nutritional therapy for diabetes mellitus. Secondary outcome: diastolic blood pressure.

Figure S15 Meta-analysis of nutritional therapy for diabetes mellitus. Secondary outcome: body mass index.

Figure S16 Funnel plot assessing publication bias on the meta-analysis of nutritional therapy for diabetes mellitus for the primary outcome (i.e.HbA1c)

Table S1 Search Strategy PubMed

#1	"Diabetes Mellitus, Type 2"[Mesh] OR (Diabetes Mellitus, Noninsulin-Dependent) OR (Diabetes Mellitus, Ketosis Resistant) OR (Ketosis-Resistant Diabetes Mellitus) OR (Diabetes Mellitus, Non Insulin Dependent) OR (Diabetes Mellitus, Non-Insulin-Dependent) OR (Non-Insulin-Dependent Diabetes Mellitus) OR (Diabetes Mellitus, Stable) OR (Stable Diabetes Mellitus) OR (Diabetes Mellitus, Type II) OR(NIDDM) OR (Diabetes Mellitus, Noninsulin Dependent) OR (Diabetes Mellitus, Maturity-Onset) OR (Diabetes Mellitus, Maturity Onset) OR (Maturity-Onset Diabetes Mellitus) OR (Maturity Onset Diabetes Mellitus) OR (MODY) OR (Diabetes Mellitus, Slow-Onset) OR (Diabetes Mellitus, Slow Onset) OR (Slow-Onset Diabetes Mellitus) OR (Type 2 Diabetes Mellitus) OR (Noninsulin-Dependent Diabetes Mellitus) OR (Noninsulin Dependent Diabetes Mellitus) OR (Maturity-Onset Diabetes) OR (Diabetes, Maturity-Onset) OR (Maturity Onset Diabetes) OR (Type 2 Diabetes) OR (Diabetes, Type 2) OR (Diabetes Mellitus, Adult-Onset) OR (Adult-Onset Diabetes Mellitus) OR (Diabetes Mellitus, Adult Onset)
#2	"Hypertension"[Mesh] OR (Blood Pressure, High) OR (Blood Pressures, High) OR (High Blood Pressure) OR (High Blood Pressures)
#3	"Primary Health Care"[Mesh] OR (Care, Primary Health) OR (Health Care, Primary) OR (Primary Healthcare) OR (Healthcare, Primary) OR (Primary Care) OR (Care, Primary) or "Physicians, Primary Care"[Mesh] OR (Physician, Primary Care) OR (Primary Care Physician) OR (Primary Care Physicians) OR (Healthy Primary Care) OR "Primary Care Nursing"[Mesh] OR (Care Nursing, Primary) OR (Nursing, Primary Care)
#4	"Community Health Planning"[Mesh] OR (Community Health Plannings) OR (Health Planning, Community) OR (Health Plannings, Community) OR (Planning, Community Health) OR (Plannings, Community Health) OR (Population-Based Planning) OR (Planning, Population-Based) OR (Plannings, Population-Based) OR (Population Based Planning) OR (Population-Based Plannings) OR (Community Health Systems) OR (Community Health System) OR (Health System, Community) OR (Health Systems, Community) OR (System, Community Health) OR (Systems, Community Health)
#5	"Nutrition Therapy"[Mesh] OR (Medical Nutrition Therapy) OR () OR (Therapy, Nutrition) OR (Nutrition Therapy, Medical) OR (Therapy, Medical Nutrition) OR (Nutrition Management) OR (Nutrition Strategy) OR (Nutrition Strategies) OR "Nutritionists"[Mesh] OR (Nutritionist) OR (Dieticians) OR (Dietician) OR (Dietitians) OR (Dietitian) OR (Registered Dietitian)
#6	"Life Style" [Mesh] OR (Life Styles) OR (Lifestyle) OR (Lifestyles) OR (Life Style Induced Illness) OR (Life Style Intervention) OR (Life Style Interventions) OR (Change of Life Style) OR "Health Behavior"[Mesh] OR (Behavior, Health) OR (Behaviors, Health) OR (Health Behaviors) OR "Healthy Lifestyle"[Mesh] OR (Lifestyle, Healthy) OR (Lifestyles, Healthy) OR (Healthy Life Styles) OR (Healthy Lifestyles) OR (Healthy Life Style) OR (Life Style, Healthy) OR (Life Styles, Healthy)
#7	randomized controlled trial[pt] OR controlled clinical trial[pt] OR randomized[tiab] OR placebo[tiab] OR clinical trials as topic[mesh:noexp] OR randomly[tiab] OR trial[ti] NOT (animals[mh] NOT humans [mh])
#1 OR #2 AND #3 OR #4 AND #5 OR #6 AND #7	

Table S2. Excluded studies and main reason for exclusion

Study	Reason for exclusion
**Diabetes Mellitus**	
Mattei da Silva 2020	Cointervention not common to both groups
Andrews 2011	Intervention not delivered in primary care setting
Absetz 2007	Patients did not meet eligibility criteria
Acik 2005	Non-randomized
Alfawaz 2018	Patients did not meet eligibility criteria
Alghafri 2018	Wrong intervention
Allen 2011	Wrong intervention
Alonso-Dominguez 2019	Wrong intervention
Al-Shookri 2011	Intervention not delivered in primary care setting
Azami 2018	Intervention not delivered in primary care setting
Babazono 2007	Patients did not meet eligibility criteria
Bowen 2016	Intervention inferior to 4 months
Carrasquillo 2017	Wrong comparator
Carter 2010	Wrong intervention
Chai 2018	Intervention not delivered in primary care setting
Chee 2017	Wrong comparator
Clark 2004	Intervention inferior to 4 months
Cohen 2011	Wrong outcome
Cooper 2008	Intervention not delivered in primary care setting
Davies 2007	Intervention inferior to 4 months
Debussche 2012	Intervention not delivered in primary care setting
Debussche 2017	Intervention not delivered in primary care setting
Delahanty 2015	Wrong comparator
Espeland 2014	Wrong comparator
Evans 2010	Wrong intervention
Two Feathers 2005	Non-randomized
Gagliardino 2013	Wrong outcome
Gallagher 2013	Intervention not delivered in primary care setting
Gregg 2016	Intervention not delivered in primary care setting
Guo 2013	Intervention not delivered in primary care setting
Gurka 2006	Intervention not delivered in primary care setting
Hardeman 2014	Wrong intervention
Hendricks 2000	Wrong comparator
Hermanns 2017	Intervention not delivered in primary care setting
Ishani 2011	Intervention not delivered in primary care setting
Janssen 2009	Intervention inferior to 4 months
Ji 2019	Intervention not delivered in primary care setting
Johansen 2017	Intervention not delivered in primary care setting
Johnson 2016	Non-randomized
Johnston 2014	Intervention not delivered in primary care setting
Kattelmann 2009	Intervention not delivered in primary care setting
Kempf 2018	Intervention not delivered in primary care setting
Khunti 2012	Intervention inferior to 4 months
Kumanyika 2018	Patients did not meet eligibility criteria
Laitinen 1993	Intervention not delivered in primary care setting
Li 2016	Intervention inferior to 4 months
Lin 2014	Patients did not meet eligibility criteria

Maindal 2012	Intervention inferior to 4 months
Merakou 2015	Intervention inferior to 4 months
Mohamed 2013	Intervention inferior to 4 months
Naik 2011	Wrong comparator
Prezio 2013	Cointervention not common to both groups
Ridgeway 1999	Intervention not delivered in primary care setting
Sarkadi 2004	Intervention not delivered in primary care setting
Shah 2015	Wrong comparator
Shakibazadeh 2015	Intervention not delivered in primary care setting
Sharifirad 2013	Intervention not delivered in primary care setting
Toobert 2003	Intervention not delivered in primary care setting
Trento 2008	Intervention not delivered in primary care setting
Philis-Tsimikas 2011	Intervention inferior to 4 months
Van Rooijen 2010	Intervention not delivered in primary care setting
Yannakoulia 2008	Intervention not delivered in primary care setting
Zhang 2018	Intervention not delivered in primary care setting
**Hypertension**	
Appel 2003	Intervention not delivered in primary care setting
Benfari 1981	Cointervention not common to both groups
Benner 2007	Wrong intervention
Ferrara 2012	Intervention not delivered in primary care setting
Friedberg 2015	Intervention not delivered in primary care setting
Fuchs 1993	Intervention inferior to 4 months
Gorostegi-Anduaga 2018	Intervention not delivered in primary care setting
Hasandokht 2015	Intervention inferior to 4 months
Hayashi 2010	Patients did not meet eligibility criteria
Heisler 2012	Wrong intervention
Jehn 2006	Wrong outcome
Kucharksa 2017	Intervention not delivered in primary care setting
Lien 2007	Intervention not delivered in primary care setting
Meinert 1989	Patients did not meet eligibility criteria
Mühlhauser 1993	Intervention inferior to 4 months
New 2003	Intervention not delivered in primary care setting
Nguyen-Huynh 2019	Study Protocol
Perl 2016	Intervention inferior to 4 months
Sookaneknun 2004	Intervention not delivered in primary care setting
Ter Bogt 2001	Patients did not meet eligibility criteria
Turnbull 2006	Wrong intervention
Wong 2015	Wrong intervention

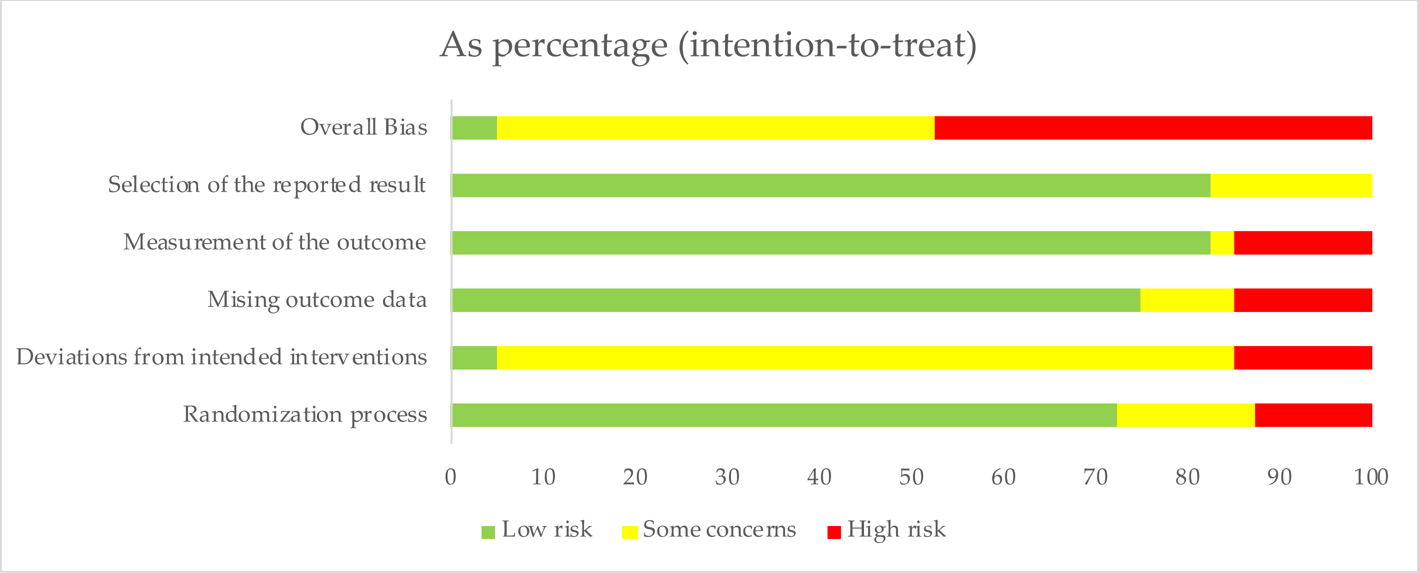

Figure S1. Summary of risk of bias

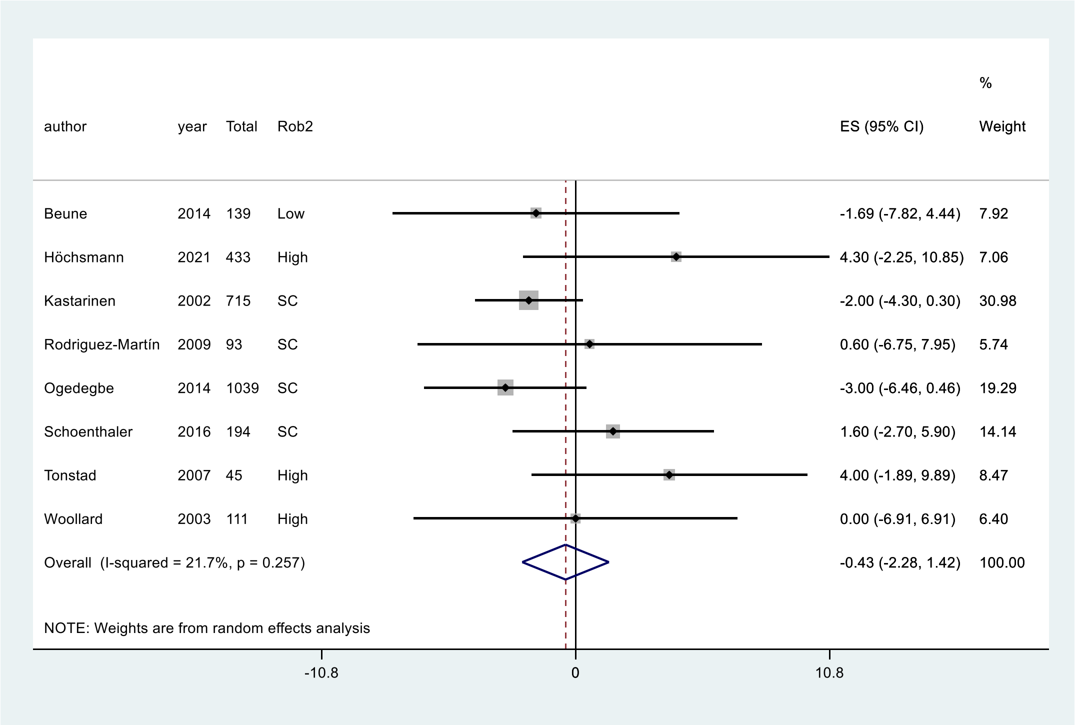

A

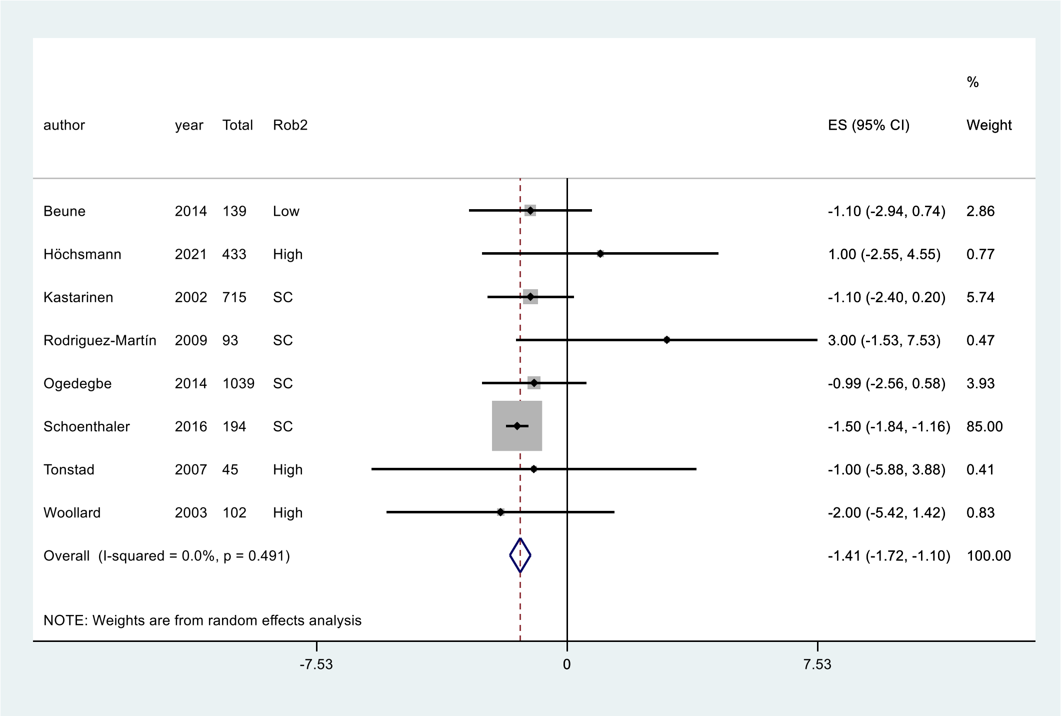

B

Figure S2 Meta-analysis of nutritional therapy for hypertension, sensitivity analysis without Hacihasanoğlu. **A**. effect on systolic blood pression, **B**. effect on diastolic blood pressure.

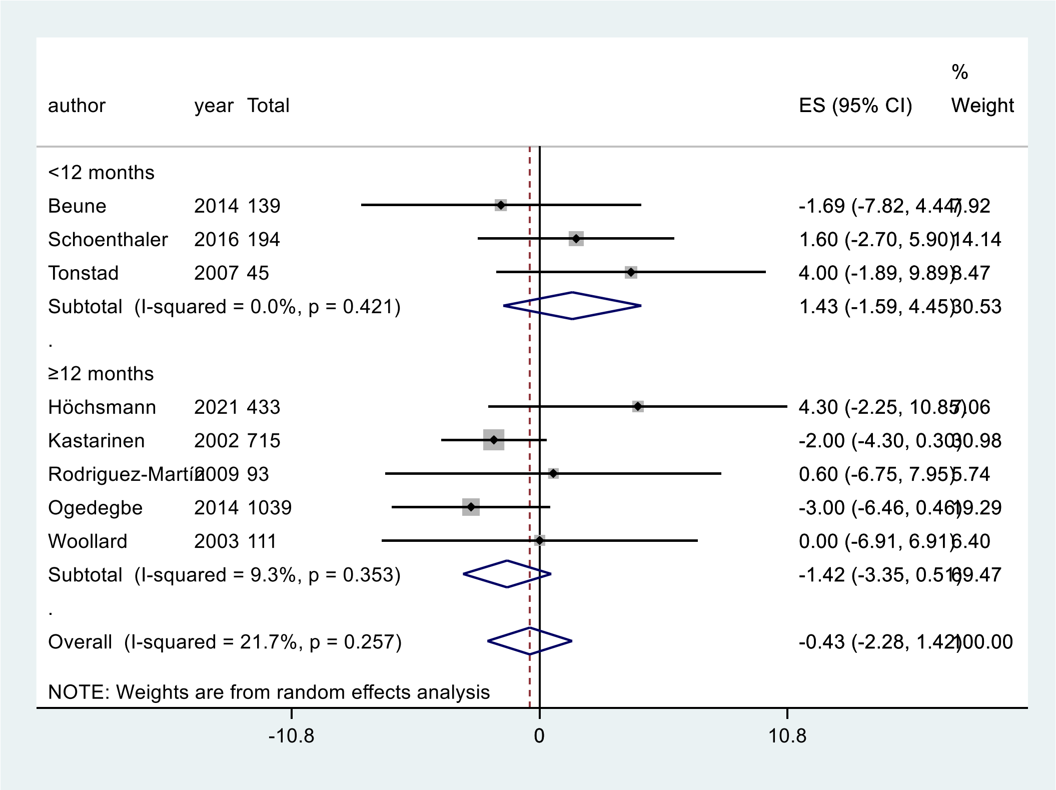

Figure S3 Meta-analysis of nutritional therapy for hypertension for primary outcome (systolic blood pressure), subgroup analysis according to length of follow-up.

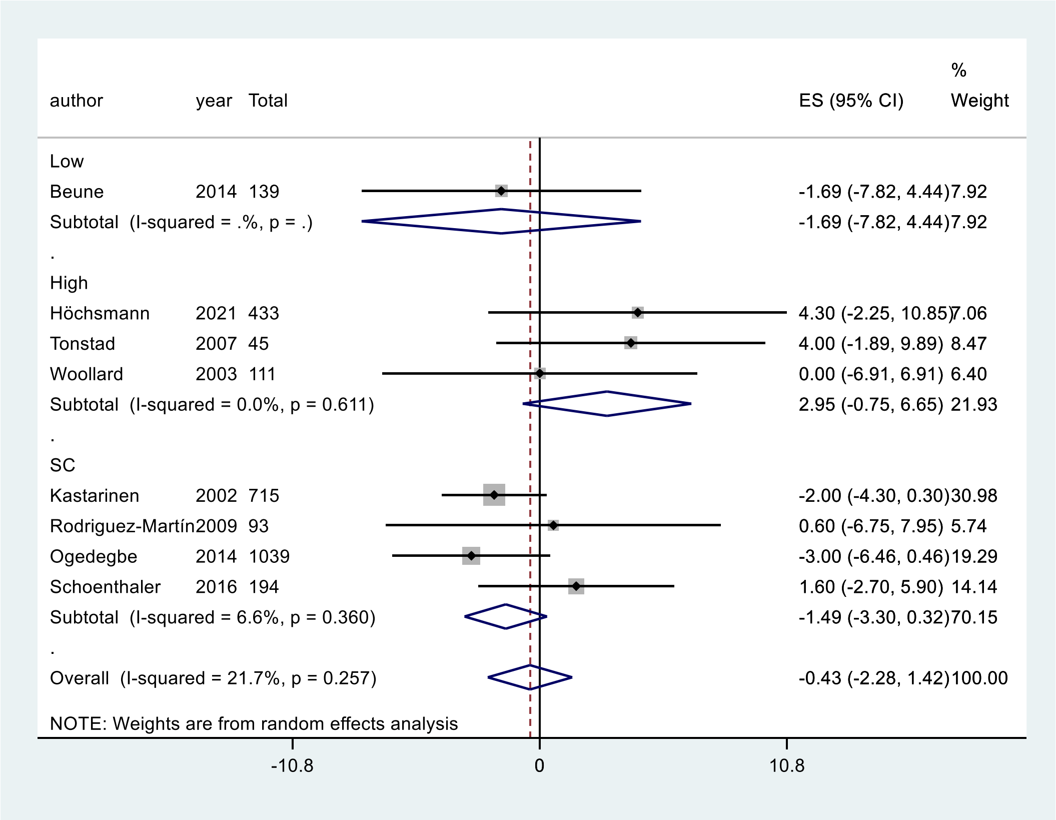

Figure S4 Meta-analysis of nutritional therapy for hypertension for primary outcome (systolic blood pressure), subgroup analysis according to risk of bias. Abbreviations: SC: some concerns.

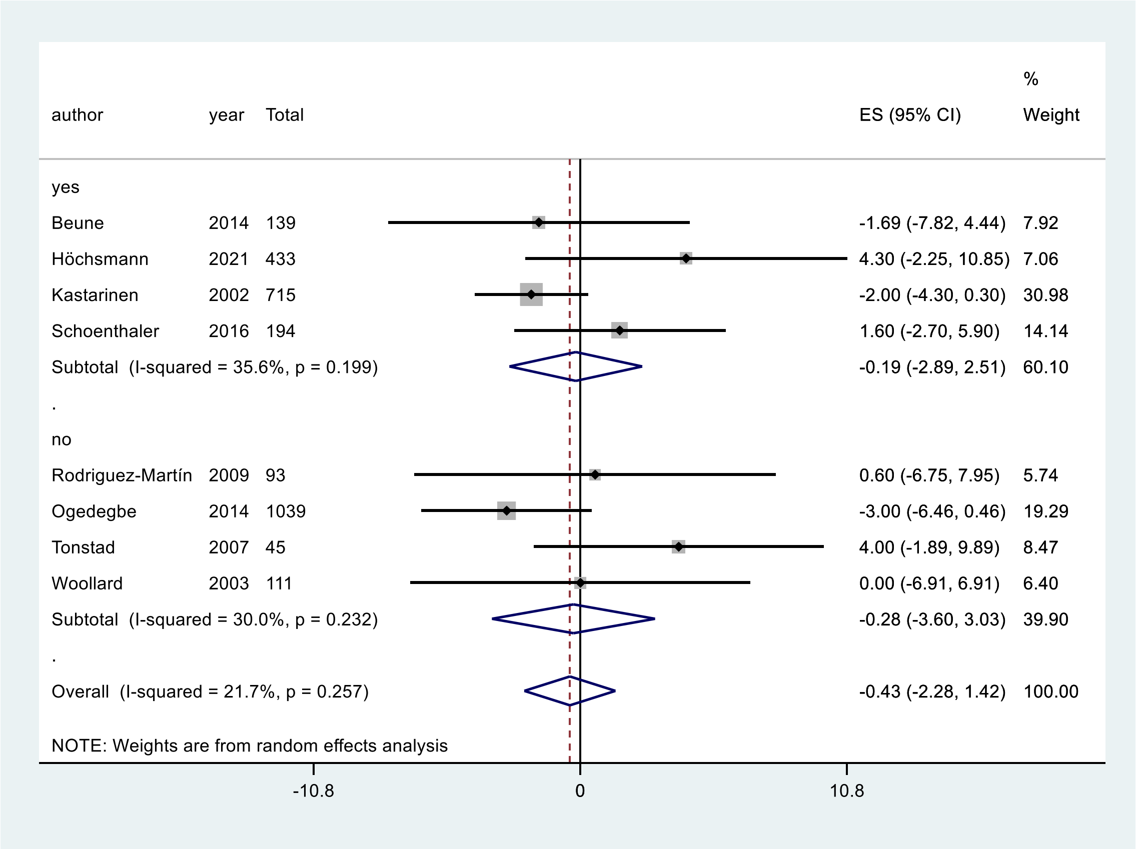

Figure S5 Meta-analysis of nutritional therapy for hypertension for primary outcome (systolic blood pressure) separating studies that presented the results as mean adjusted difference (i.e. change score) or post-intervention values.

```
. xi:metareg    md dietic rob madcs follow, wsse(se)

Meta-regression                                       Number of obs  =       27
REML estimate of between-study variance               tau2           =    .1772
% residual variation due to heterogeneity             I-squared_res  =   86.59%
Proportion of between-study variance explained        Adj R-squared  =   -6.67%
Joint test for all covariates                         Model F(4,22)  =     0.58
With Knapp-Hartung modification                       Prob > F       =   0.6775

------------------------------------------------------------------------------
          md | Coefficient  Std. err.      t    P>|t|     [95% conf. interval]
-------------+----------------------------------------------------------------
      dietic |    .126805   .2296136     0.55   0.586    -.3493846    .6029945
         rob |  -.0545942   .2061508    -0.26   0.794    -.4821248    .3729363
       madcs |  -.0510415   .1986437    -0.26   0.800    -.4630033    .3609202
      follow |   .2536292   .2190916     1.16   0.259    -.2007389    .7079973
       _cons |   -.589575   .3252521    -1.81   0.084    -1.264107    .0849566
------------------------------------------------------------------------------
```

Figure S6 Meta-regression assessing follow up as modifier of the effect of nutritional therapy on HbA1c

```
. metareg    md meanage meanhba1c , wsse(se)

Meta-regression                                         Number of obs  =      26
REML estimate of between-study variance                 tau2           =   .1554
% residual variation due to heterogeneity               I-squared_res  =  81.99%
Proportion of between-study variance explained          Adj R-squared  =  10.04%
Joint test for all covariates                           Model F(2,23)  =    1.80
With Knapp-Hartung modification                         Prob > F       =  0.1881

------------------------------------------------------------------------------
          md | Coefficient  Std. err.      t    P>|t|     [95% conf. interval]
-------------+----------------------------------------------------------------
     meanage |   .0285371   .0243296     1.17   0.253    -.0217925    .0788667
   meanhba1c |  -.0583827   .0694295    -0.84   0.409    -.2020085    .0852431
       _cons |  -1.574059   1.746094    -0.90   0.377     -5.18613    2.038012
------------------------------------------------------------------------------
```

Figure S7 Meta-regression assessing mean age and mean HbA1cs as modifiers of the effect of nutritional therapy on HbA1c

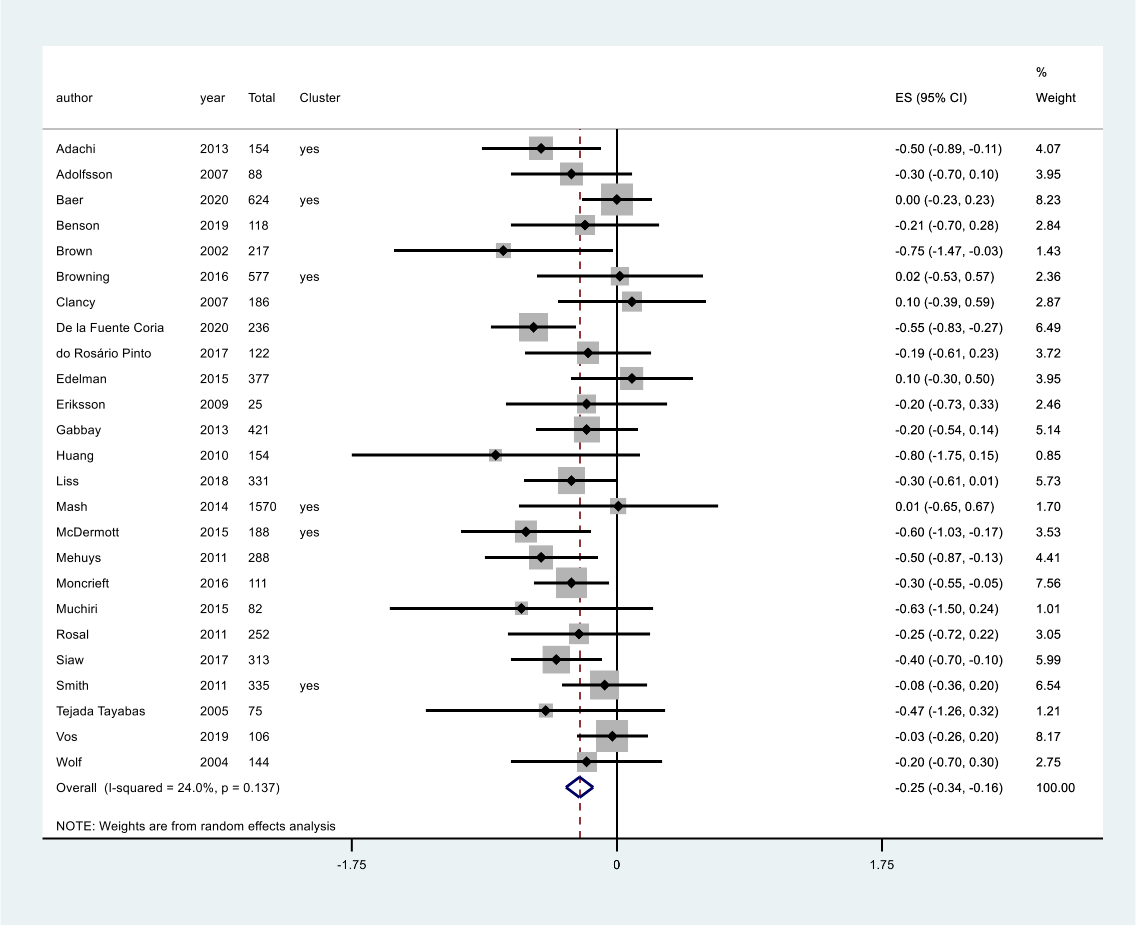

Figure S8 Meta-analysis of nutritional therapy for diabetes mellitus for primary outcome (HbA1c) without Javaid et al and Hörnstein et al.

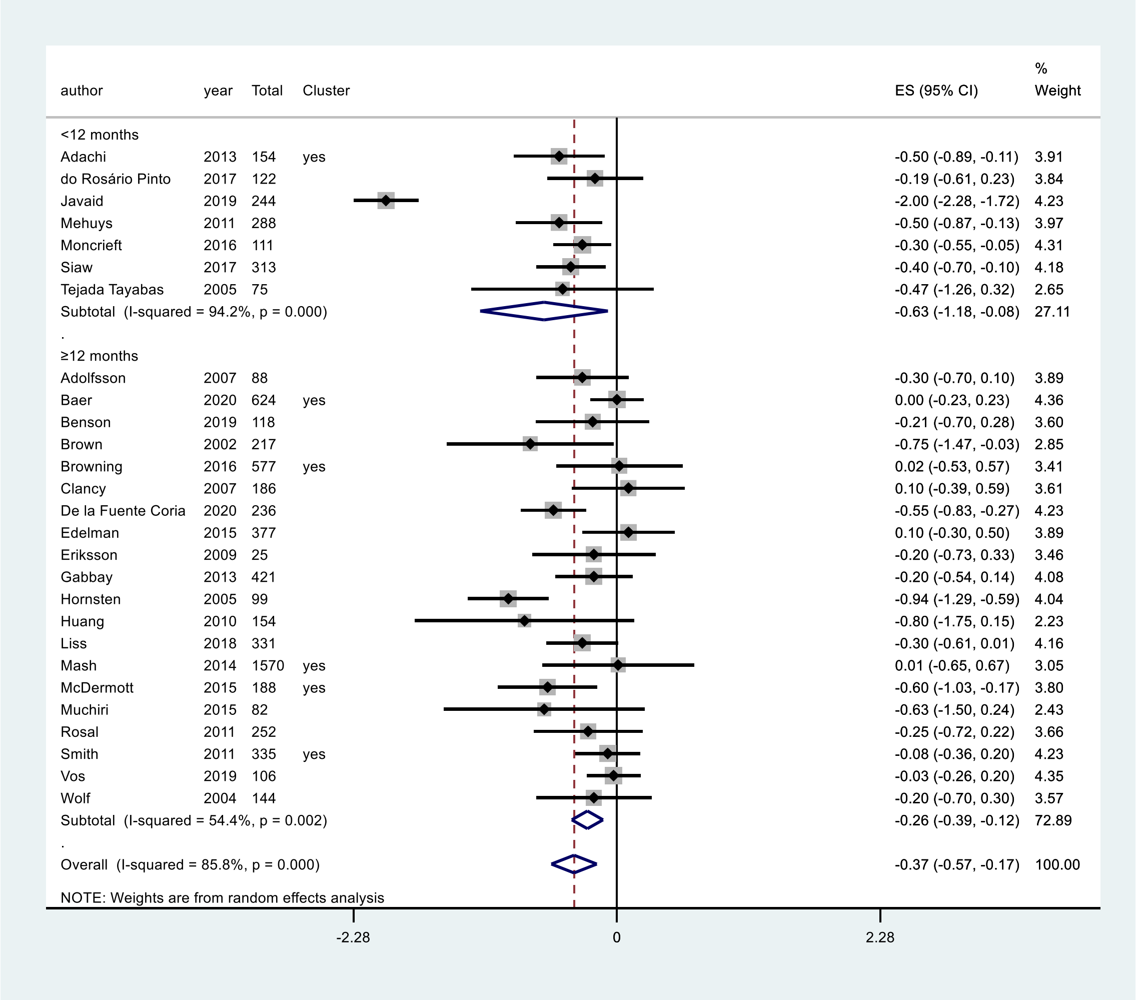

Figure S9 Meta-analysis of nutritional therapy for diabetes mellitus for primary outcome (HbA1c), subgroup analysis according to length of follow up.

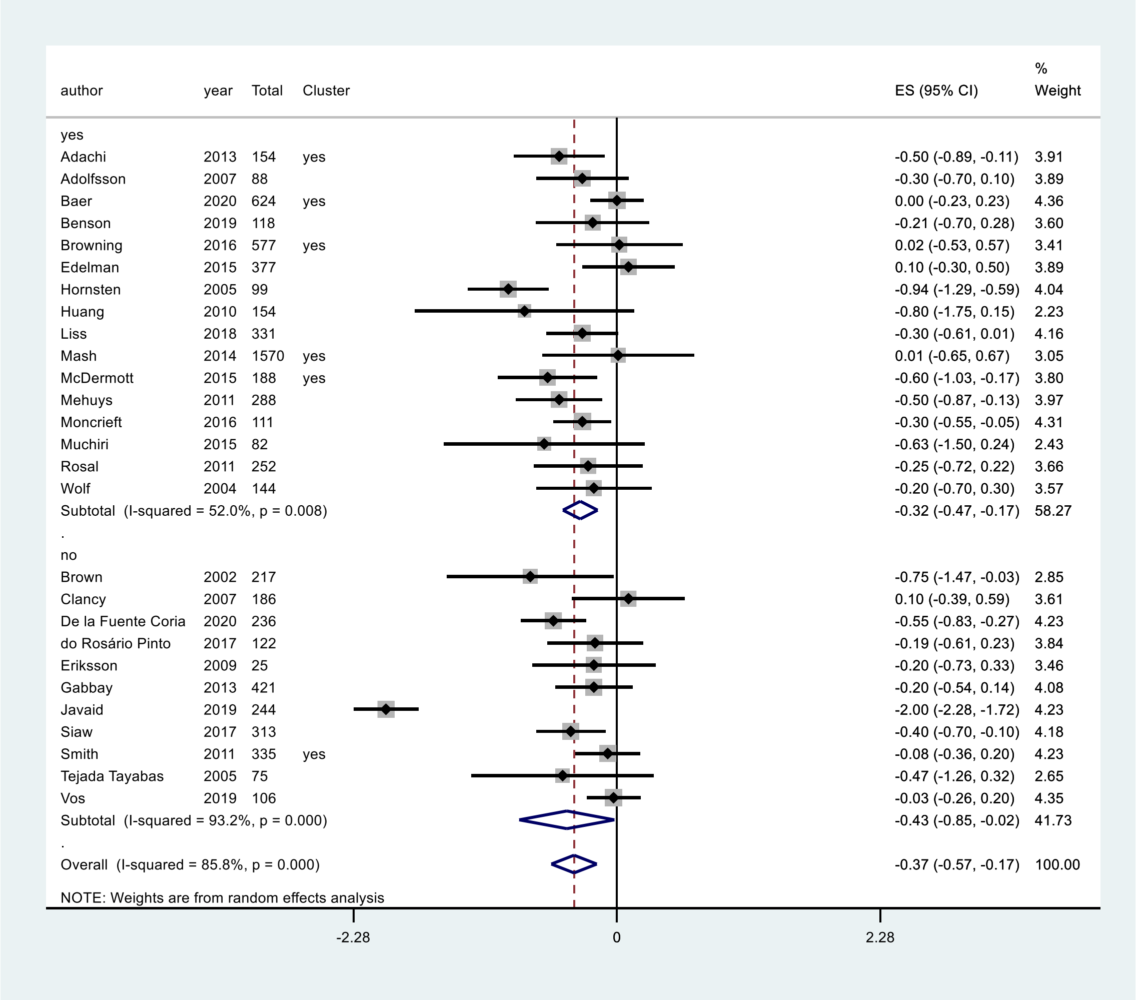

Figure S10 Meta-analysis of nutritional therapy for diabetes mellitus for primary outcome (HbA1c), subgroup analysis separating studies that presented the results as mean adjusted difference (i.e. change score) or post-intervention values.

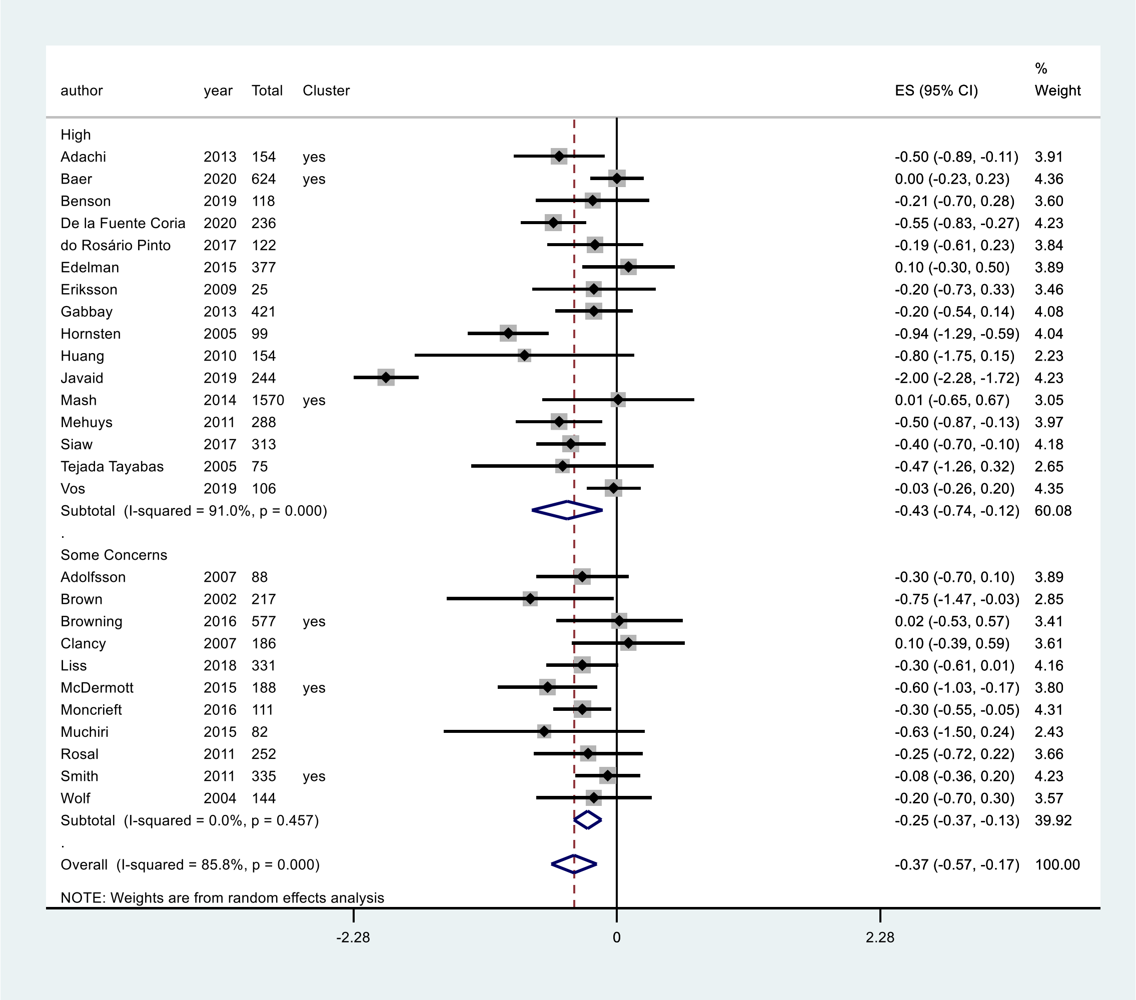

Figure S11 Meta-analysis of nutritional therapy for diabetes mellitus for primary outcome (HbA1c), subgroup analysis according to risk of bias.

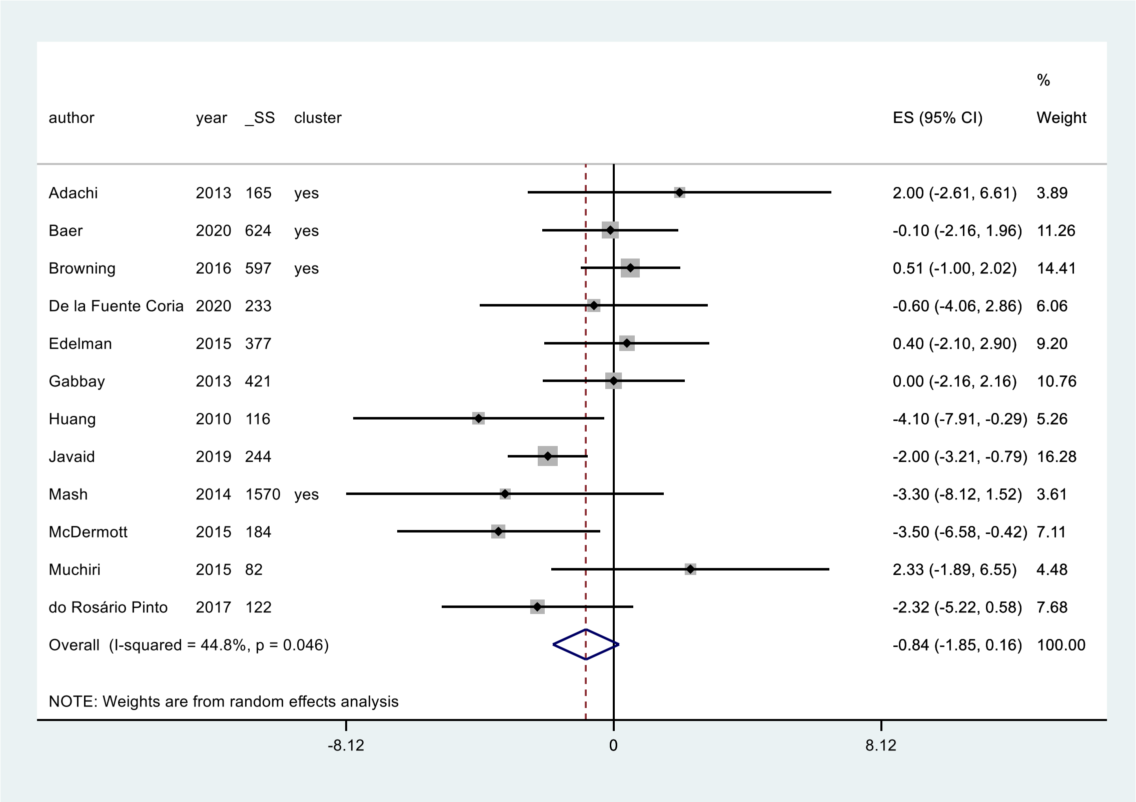

Figure S12 Meta-analysis of nutritional therapy for diabetes mellitus. Secondary outcome: diastolic blood pressure.

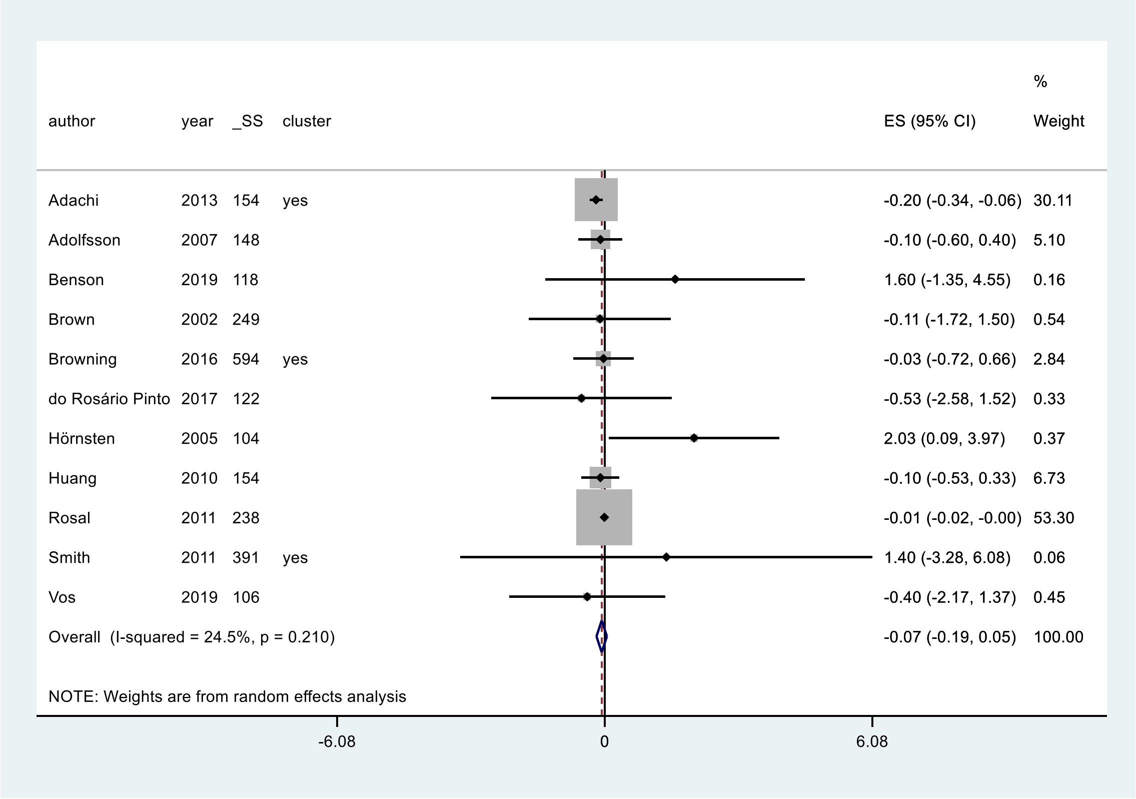

Figure S13 Meta-analysis of nutritional therapy for diabetes mellitus. Secondary outcome: body mass index.

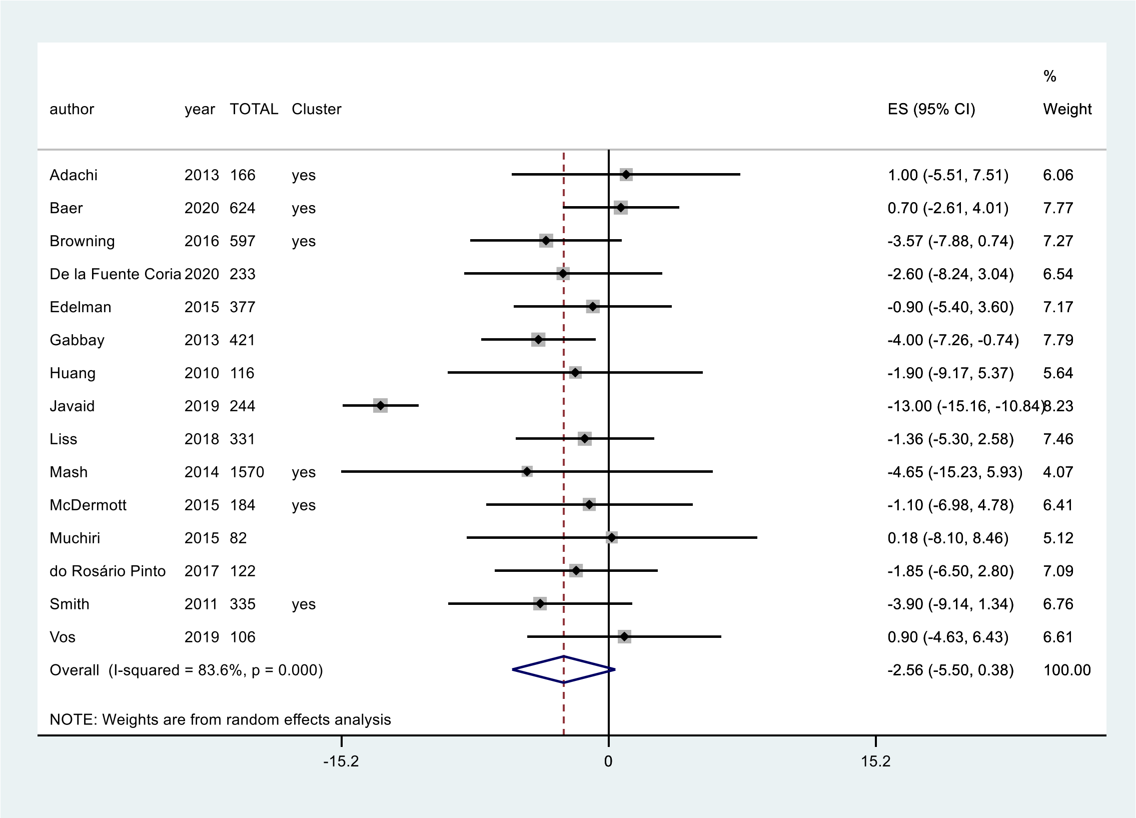

Figure S14 Meta-analysis of nutritional therapy for diabetes mellitus. Secondary outcome: systolic blood pressure.

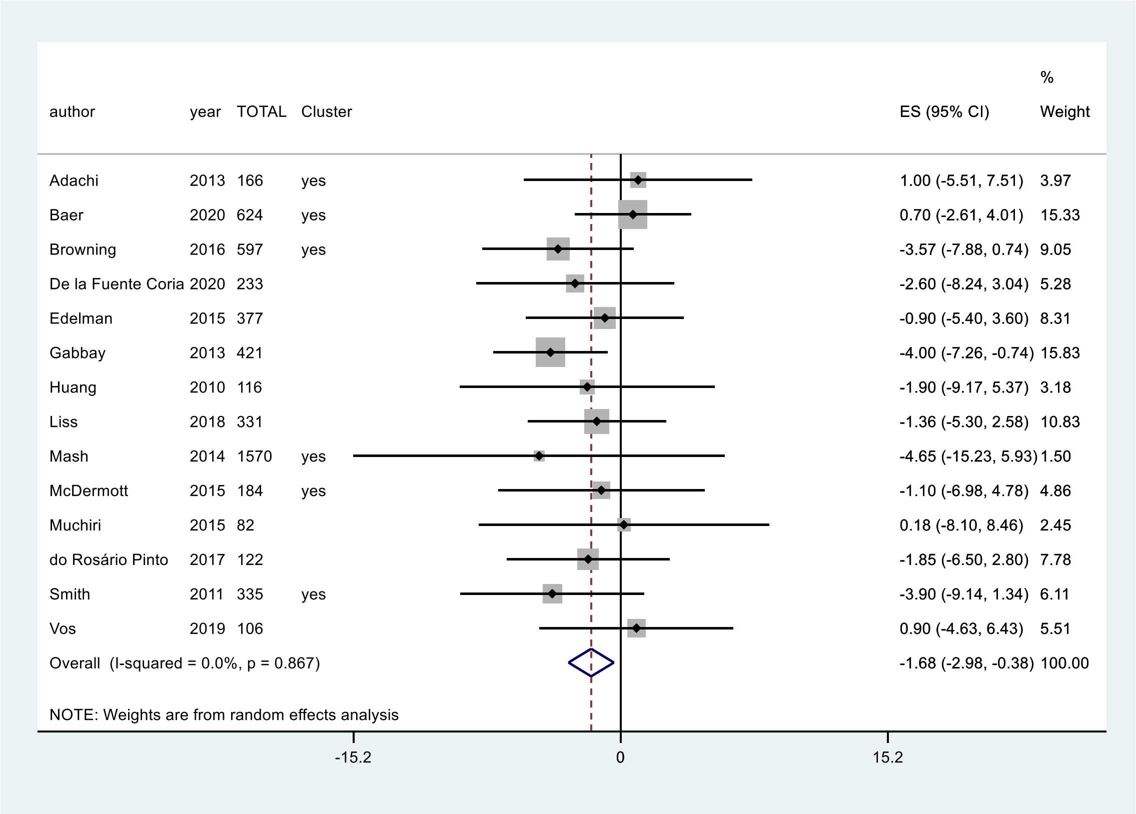

Figure S15 Meta-analysis of nutritional therapy for diabetes mellitus without Javaid et al. Secondary outcome: systolic blood pressure.

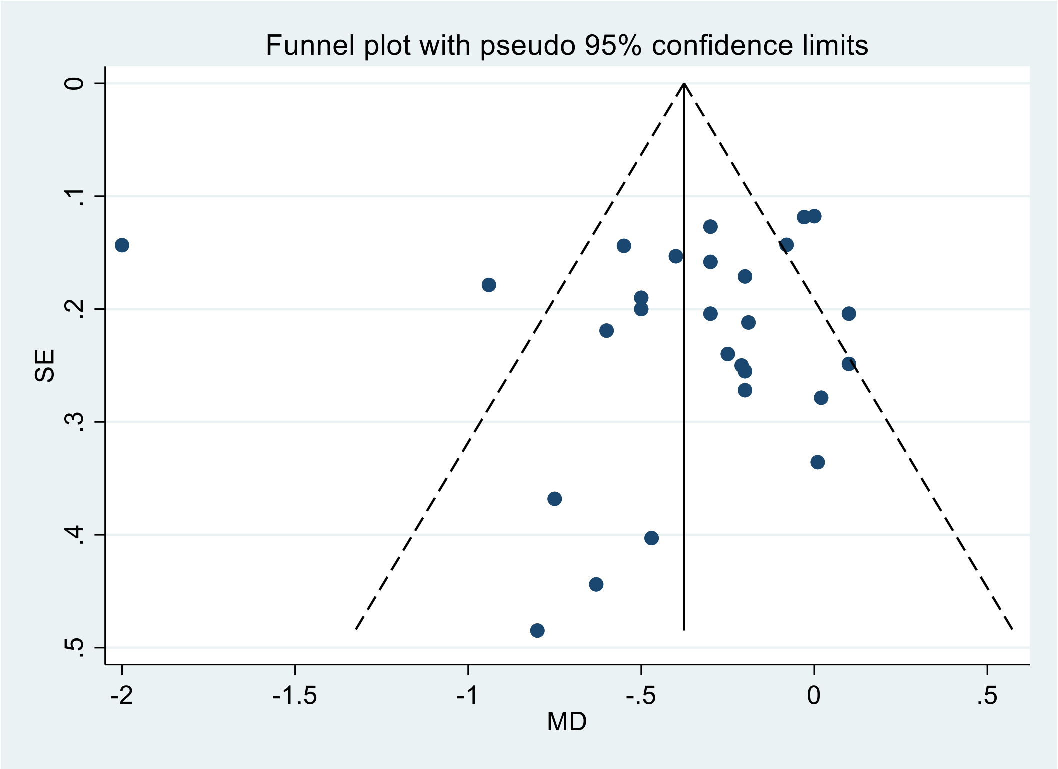

Figure S16. Funnel plot assessing publication bias on the meta-analysis of nutritional therapy for diabetes mellitus for the primary outcome (i.e.HbA1c)
